# DNA Sequence Explains Seemingly Disordered Methylation Levels in Partially Methylated Domains of Mammalian Genomes

**DOI:** 10.1371/journal.pgen.1004143

**Published:** 2014-02-13

**Authors:** Dimos Gaidatzis, Lukas Burger, Rabih Murr, Anita Lerch, Sophie Dessus-Babus, Dirk Schübeler, Michael B. Stadler

**Affiliations:** 1 Friedrich Miescher Institute for Biomedical Research, Basel, Switzerland; 2 Swiss Institute of Bioinformatics, Basel, Switzerland; 3 Faculty of Science, University of Basel, Basel, Switzerland; Mount Sinai School of Medicine, United States of America

## Abstract

For the most part metazoan genomes are highly methylated and harbor only small regions with low or absent methylation. In contrast, partially methylated domains (PMDs), recently discovered in a variety of cell lines and tissues, do not fit this paradigm as they show partial methylation for large portions (20%–40%) of the genome. While in PMDs methylation levels are reduced on average, we found that at single CpG resolution, they show extensive variability along the genome outside of CpG islands and DNase I hypersensitive sites (DHS). Methylation levels range from 0% to 100% in a roughly uniform fashion with only little similarity between neighboring CpGs. A comparison of various PMD-containing methylomes showed that these seemingly disordered states of methylation are strongly conserved across cell types for virtually every PMD. Comparative sequence analysis suggests that DNA sequence is a major determinant of these methylation states. This is further substantiated by a purely sequence based model which can predict 31% (R^2^) of the variation in methylation. The model revealed CpG density as the main driving feature promoting methylation, opposite to what has been shown for CpG islands, followed by various dinucleotides immediately flanking the CpG and a minor contribution from sequence preferences reflecting nucleosome positioning. Taken together we provide a reinterpretation for the nucleotide-specific methylation levels observed in PMDs, demonstrate their conservation across tissues and suggest that they are mainly determined by specific DNA sequence features.

## Author Summary

Methylation is an essential DNA modification, which is attracting a lot of attention as a regulator of gene expression. Recent technological advances have allowed the genome-wide measurement of methylation at single-nucleotide resolution, leading to the discovery of several new types of methylation patterns. One prominent example are partially methylated domains (PMDs), which are regions with reduced average methylation, covering up to 40% of the genome. PMDs are found in only a subset of cell types, particularly in differentiated and cancer cells. An outstanding question is how methylation levels in PMDs are determined and how they can be interpreted at the single-nucleotide level. Here we provide a new model of methylation in PMDs. Single-nucleotide methylation levels in PMDs, albeit reduced on average, are highly variable along the genome. Furthermore, they are precisely set and can be predicted using DNA sequence features, establishing a new link between methylation and the underlying genetic information. This results in a high correlation of methylation levels in PMDs across different cell types. Our findings suggest that in any comparative analyses, PMDs should be analyzed as entities, strongly reducing the complexity of high-resolution DNA methylation analyses.

## Introduction

DNA methylation in metazoan genomes occurs mainly in the context of CpG dinucleotides and for the most part genomes are highly methylated and harbor only small regions with low or absent methylation [1]. These include CpG islands [1], CpG island shores [2] and distal regulatory regions [3], [4]. Partially methylated domains (PMDs) depart from this notion. They were initially discovered through whole-genome bisulfite sequencing in human fibroblasts [5] and correspond to large genomic domains (mean = 153 kb) with average methylation levels of less than 70%, covering almost 40% of the genome. Interestingly these domains were not detected in H1 human embryonic stem cells. The PMDs in IMR90 were shown to harbor genes with reduced expression, to correlate with repressive and to anti-correlate with active histone marks. In a subsequent study PMDs were detected in three additional cell types, namely foreskin fibroblasts (FF), adipose-derived stem cells (ADS) and adipocytes (ADS-adipose) [6]. While every cell type showed specific patterns of PMD localization, overall a high fraction of PMDs co-localized in all cell types. On induction of pluripotency, the presence of PMDs was strongly reduced in all the cell types examined. In later studies, PMDs were found in SH-SY5Y neuronal cells [7], human mammary epithelial cells [8] as well as colorectal cancer samples where they were shown to coincide with late replicating and nuclear lamina associated regions [9]. Very recently and importantly, human placenta was shown to contain PMDs, representing the first known uncultured and non-cancer tissue type with PMDs [10].

Understanding methylation levels in PMDs is of great interest as they represent a widespread methylation pattern that is distinct from the reduced methylation state of CpG islands [1] and at DHS [3], [4], [11], which are indicative of transcription factor binding [11], or the fully-methylated state in most of the genome. Additionally, PMDs may be directly linked to hypomethylation that is frequently observed in cancer [8], [12], [13]. Studies on PMDs so far focused mainly on their macroscopic properties, their genomic locations and conditions in which they are formed or abolished. In this study we set our main focus on the microscopic aspects of PMDs, investigating how this phenomenon manifests at the level of single nucleotides. We show that single-CpG methylation levels in PMDs, in contrast to the remainder of the genome, are roughly uniformly distributed, spanning the entire range from 0 to 100 percent methylation, and display a seemingly disordered pattern along the genome. Surprisingly, these patterns are conserved across PMD-containing cell types and sequence analysis suggests that the DNA sequence itself is a major determinant of their methylation states. A position-specific dinucleotide model reveals CpG density and various dinucleotides immediately flanking the CpG as the main drivers of methylation levels in PMDs. Importantly these sequence features are specific to PMDs as they contribute only little to the methylation levels in the remainder of the genome.

## Results

### CpG methylation levels in PMDs are highly variable along the genome

**Figure 1:**
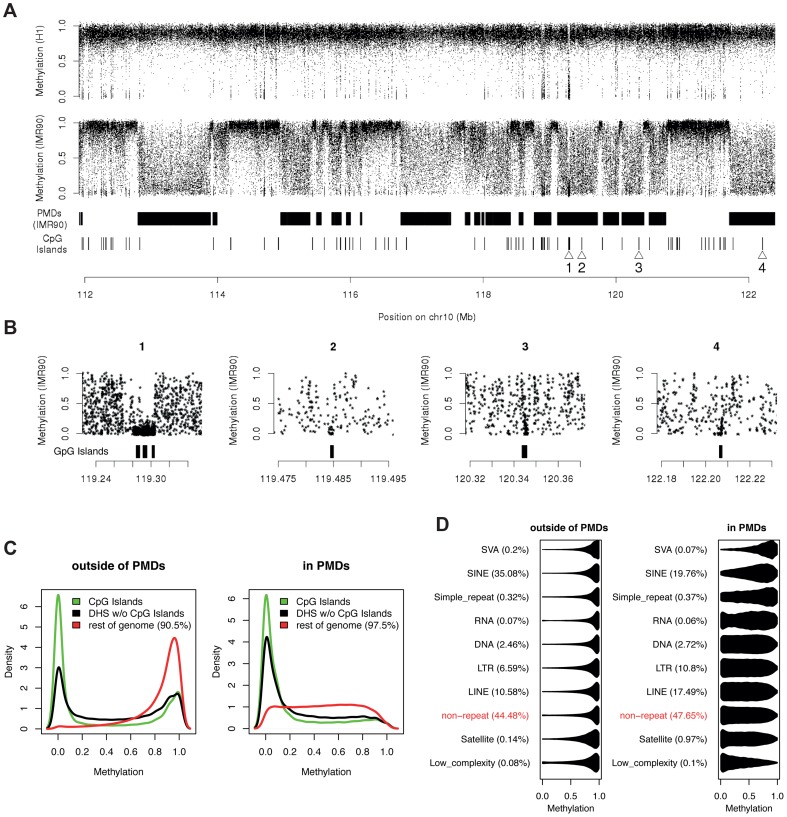
CpG methylation levels in PMDs are highly variable along the genome. (a) Methylation status of single CpGs in one genomic region covering 10% of chromosome 10 (hg18 human genome assembly) for the cell types H1 and IMR90. One dot represents one CpG, the y-axis denotes percentage methylation (both strands combined). Only CpGs covered by at least 10 reads are shown. Methylation levels were jittered by +−2% to reduce overplotting. PMDs [5] and CpG islands are shown as black rectangles. The numbered triangles indicate example CpG islands illustrated in (b) in an expanded view. (c) Genome-wide methylation level distributions of single CpGs in CpG islands, DHS outside of CpG islands and the rest of the genome, both inside and outside of PMDs, shown for all autosomal CpGs covered by at least 10 reads in IMR90. (d) Genome-wide distribution of methylation levels of CpGs classified by repeat annotation. Only repetitive elements with at least 1000 CpGs are shown.

So far, the main feature that has been used to characterize and localize PMDs is reduced mean methylation calculated over large windows containing many CpGs [5], [6], [7], [9]. To investigate the phenomenon on the single nucleotide level, we visualized the methylation status of single CpGs in one genomic region covering 10% of chromosome 10 (Figure 1a). In human embryonic stem cells (H1), high methylation is predominant with a relatively small number of hypomethylated regions. In IMR90 fibroblasts however, the situation is dramatically different. There exist distinct domains with clear boundaries that display aberrant methylation levels. On average these domains exhibit decreased (partial) methylation levels. Importantly however this decrease in average methylation is not a result of a constant overall reduction at all CpGs. It is mainly attributed to an extensive increase in the variability in methylation along the genome. Single CpG methylation levels in PMDs span the full range from 0% to 100% in a roughly uniform fashion (Figure 1a). Due to this high variability PMDs appear as domains of highly disordered methylation. However this is not a reflection of a random process at the single cell level. The methylation level for a given CpG refers to the percentage of alleles carrying the methyl group in a population of cells, thus a randomly established methylation state at the single cell level would average out to a methylation level of 50% for each CpG. This is not in accordance with the data and therefore strongly argues that a mechanism must exist which acts in individual cells and determines the likelihood of methylation. In particular this likelihood needs to be CpG-dependent.

It is well known that DNA methylation is generally reduced at CpG islands [1]. More recently this has also been shown to be the case for DHS outside of CpG islands [3], [4]. To investigate to what extent the variability of methylation levels in PMDs in IMR90 can be attributed to the presence of these regions, we determined the genome-wide distribution of methylation levels for CpGs within CpG islands, DHS and the remaining ones within PMDs. As a contrast, we performed the same analysis for CpGs outside of PMDs, which display the typical well-known patterns of mammalian methylomes (Figure 1a). The majority of CpG islands and DHS in PMDs show reduced methylation, similar to what is observed outside of PMDs (Figure 1b and c). The remaining CpGs, which constitute the large majority of CpGs in PMDs, however, display a roughly uniform distribution in methylation levels. This is in stark contrast to the situation outside of PMDs, where the large majority of CpGs outside of islands and DHS are fully methylated. It thus appears that unlike outside of PMDs, most of the variability in methylation within PMDs cannot be attributed to the presence of CpG islands nor DHS. For all the subsequent analyses, we therefore considered only the CpGs outside of CpG islands and, if available, outside of DHS.

PMDs have been shown to be located predominantly in heterochromatic, gene-poor regions [5], which are rich in repeat elements. To investigate the relationship between the presence of particular repeat elements and methylation levels in PMDs, we grouped CpGs according to repeat annotation and monitored their distribution of methylation levels (Figure 1d). In contrast to repeat elements outside of PMDs, which are generally fully methylated, repeats inside PMDs do not maintain the fully-methylated state and show markedly increased variability, comparable to non-repeat regions. SVAs (composite unit of SINE, VNTR and *Alu* [14]), which make up only a small fraction of the genome, constitute an exception as they partially maintain their fully methylated state. SVAs are known to be active and are generally methylated [15], [16], but the functional significance of this methylation is still unclear[17]. We conclude that the variability of methylation levels in PMDs cannot simply be explained by the preferential methylation levels of particular repeat types and their distribution along the genome.

### Levels of CpG methylation in PMDs are conserved across cell types

**Figure 2:**
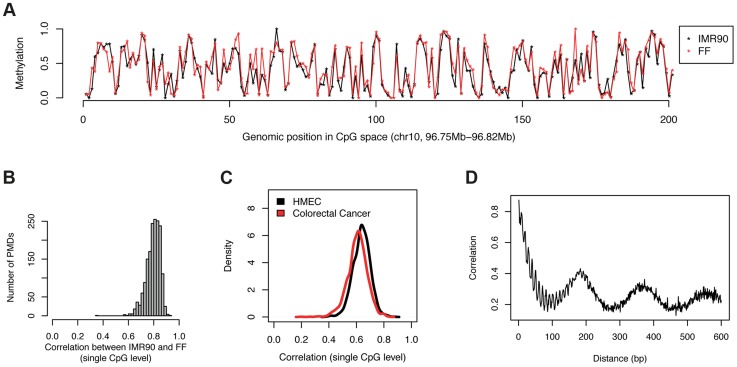
Levels of CpG methylation in PMDs are conserved across cell types. (a) Example profile illustrating the similarity of single CpG methylation levels between IMR90 and FF. For better visibility, CpGs have been drawn at equal distance from each other. (b) For each PMD present in IMR90 and FF, we quantified the similarity in the single CpG methylation levels between the two tissues using the pearson correlation coefficient. The histogram shows the distributions of all the correlation coefficients (one for each PMD). (c) Same as b), but comparing IMR90 to HMEC as well as colorectal cancer cells. (d) Distance-dependent correlation of the methylation levels of adjacent CpGs in PMDs (IMR90).

To gain more insight into methylation levels in PMDs, we compared single CpG methylation levels in PMDs across different PMD-containing tissues. To identify PMDs across methylomes, we previously developed an algorithm which makes direct use of the uniformity of methylation levels in PMDs, a functionality which we provide as part of the MethylSeekR R package [18]. We identified high-confidence (see Materials and Methods) PMDs in IMR90 (covering 1.32 Gb) and separately in FF (1.36 Gb) and selected PMDs common to both cell types (1.22 Gb). A comparison of methylation levels along the genome revealed a strong agreement between the two cell types at the single CpG level (Figure 2a). To globally assess this trend, we quantified, for every PMD, the similarity between single CpG methylation levels in IMR90 and FF using the pearson correlation coefficient. Strikingly, methylation levels are conserved across the two cell types with correlation coefficients ranging from 0.7 to 0.9 (Figure 2b). This high similarity between the methylation at individual CpGs within PMDs is not confined to fibroblast tissues. A comparison of IMR90 to human mammary epithelial cells (HMEC, 1.20 Gb of PMDs, 1.03 Gb in common with IMR90) [8] revealed high correlations ranging from 0.5 to 0.7 (Figure 2c). Interestingly, the same holds true in a comparison of IMR90 to colorectal cancer cells (1.39 Gb of PMDs, 1.06 Gb in common with IMR90), which were shown to contain PMDs [9] (Figure 2c). It is important to note that in these cancer cells, it appears that CpG islands within PMDs tend to have increased rather than reduced methylation as in healthy tissue [9]. This, however, does not influence our analysis as we exclude CpGs overlapping CpG islands from our analysis.

The surprising similarity of methylation levels between PMD-containing cell-types substantiates the requirement for a mechanism that accounts for the specificity of methylation at the single CpG level. Since methylation is binary on an allele level, the process that sets the methylation mark must be stochastic in nature. For example for a CpG with a methylation level of 20% in the population, the probability of obtaining a methyl mark for a single allele must be 0.2. Where does the information come from that is required by each cell to determine this probability for every single CpG within a PMD? One likely candidate is the underlying DNA sequence or its chromatin state.

From a bird's eye view as illustrated in Figure 1a, it seems that there is no dependence of methylation levels between consecutive CpGs. However this might be solely due to the fact that CpGs are scarce and irregularly spaced. In order to account for this, we calculated correlations for consecutive CpGs in a distance–dependent manner (Figure 2d). This analysis revealed that CpGs in very close proximity (<15 nt) correlate very well (r = 0.8) but that this correlation deteriorates rapidly with distance. CpGs at the average spacing of 109 bp show only little spatial coupling (r = 0.23). Furthermore we identified a weaker signal with a periodicity of 10 bp, which is likely related to the turn of the DNA helix wrapped around the nucleosome [19]. Finally we detected a local maximum at about 180 bp [20] corresponding to typical distances between nucleosomes. Taken together this suggests that nucleosomes might be involved in the process that determines methylation levels for individual CpGs. It is known that positioning of nucleosomes is at least partially dependent on the underlying DNA sequence [21]. We therefore investigated if DNA sequence or nucleosome positioning can explain the methylation levels of single CpGs.

### DNA sequence can predict methylation levels in PMDs

**Figure 3:**
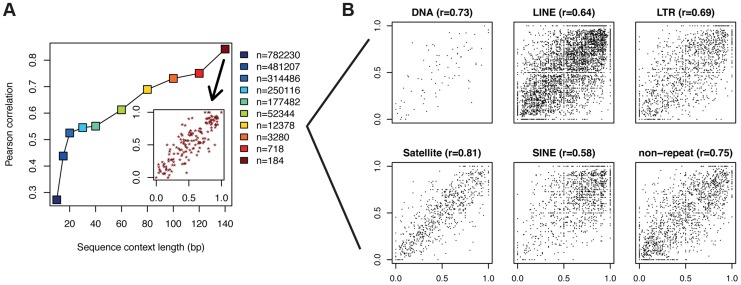
CpGs with identical flanking sequence display similar methylation. (a) For each CpG in a PMD, the surrounding sequence (CpG in the center) was extracted and used to identify sequences occurring multiple times in the genome. Correlation coefficients for pairs of CpGs with identical flanking sequences are depicted on the y-axis. This procedure was performed for various sequence lengths ranging from 10 bp to 140 bp. The inlet displays the actual data points giving rise to the correlation coefficient at 140 bp. In the case where a sequence occurred more than twice in the genome, only one randomly selected pair was considered. The legend denotes the number of pairs of data points available for each sequence length. (b) Scatter plots of methylation levels for CpG pairs with identical sequence context of length 80 (yellow square in (a)) separated by repeat annotation. Only repeat classes with at least 100 data points are shown.

To ask if DNA sequence has the potential to predict methylation levels of single CpGs in PMDs of fibroblasts, we performed two types of analyses, one based on a comparative sequence approach and one based on a nucleotide model. Firstly we addressed the question whether methylation levels tend to be conserved in CpGs with identical surrounding sequence. To do so, for each CpG in a PMD, we extracted the surrounding sequence (centered around the CpG) and grouped all the CpGs based on that sequence. This allowed us to calculate correlation coefficients from pairs of CpGs with identical flanking sequences. We performed this procedure for various sequence lengths ranging from 10 bp to a maximum of 140 bp. Longer sequences could not be studied due to the limited read lengths of 87 bp provided in the methylome. At a sequence context of 140 bp (with the CpG in the center), at most 17 bp remain to uniquely map the bisulfite read as it also needs to overlap the central CpG to quantify the methylation level. The analysis revealed that with increasing sequence context, correlation of methylation increases dramatically from r = 0.28 at 10 bp to r = 0.86 at 140 bp. (Figure 3a). This suggests that the DNA sequence plays a key role in determining exact methylation levels in PMDs. One limit of this analytic approach is that with increasing context length, preferentially those pairs of CpGs that reside within repetitive parts of the genome remain for quantification. These regions might be atypical and thus introduce a bias. To investigate this issue, we partitioned the CpG pairs according to their UCSC repeat annotation. To retain a sufficient number of pairs per category, we selected the flanking sequences of length 80 bp, which corresponds to 12K pairs of CpGs and a correlation of 0.69 (Figure 3a). Whereas, as expected, the large majority of CpG pairs overlap with repeat elements, there still remain 16.4% of CpG pairs that lie outside of repeats which we analyzed separately. This revealed that even within each annotation class, similar sequences exhibit similar methylation levels. In particular, there is no substantial difference between repeat and non-repeat sequences (Figure 3b).

**Figure 4:**
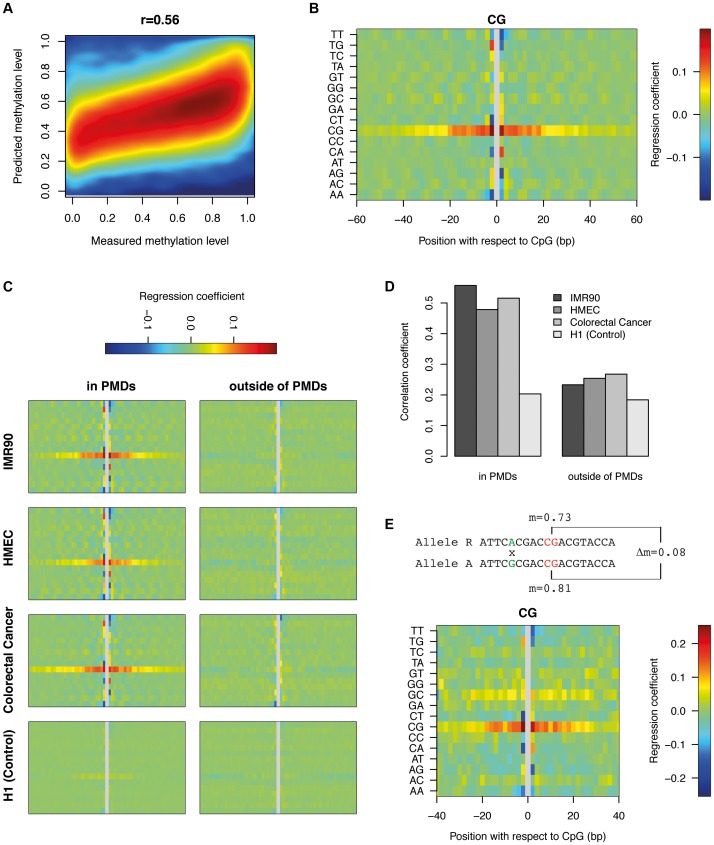
DNA sequence can predict methylation levels in PMDs in IMR90. (a) Performance of the positional dinucleotide model (see Materials and Methods) in PMDs. Depicted is the 2D-density for a total of 100K CpGs in PMDs. The x-axis denotes the measured methylation level and the y-axis denotes the predicted methylation level. (b) Inferred parameters of the positional dinucleotide model (see Materials and Methods) which predicts methylation levels in PMDs. The contributions of the 16 dinucleotides within a window of +−60 bp around the central CpG are color coded and displayed as a heatmap. (c) Same as (b) for the indicated tissues, separately for PMDs and regions outside of PMDs. For H1, which does not contain PMDs, the PMD annotation of IMR90 was used. In contrast to (b), CpGs overlapping DHS were not removed as the necessary DHS dataset were not available for all cell types. Thus panel b) and the upper left panel in c) only differ with respect to the inclusion or exclusion, respectively, of CpGs based on DHS. (d) Performance of the dinucleotide model for the same tissues and regions as in (c). (e) Contribution of each dinucleotide to allelic methylation differences in PMDs. The sketch at the top depicts the investigated genomic configuration, namely a CpG conserved in both alleles but flanked by a SNP. The difference of one nucleotide is used to assign the bisulfite reads covering the SNP to an allele (Reference or Alternative). The reads that cover both, the SNP as well as the conserved CpG are used to quantify the allele specific methylation level of the conserved CpG. Given the nucleotides involved in the SNP and the difference in methylation level between the two alleles, we calculated the allele specific contribution of each dinucleotide at a given distance from the conserved CpG (see Materials and Methods). The analysis was performed within a window of +−40 bp due the low number of data points available at larger distances.

To characterize the role of sequence features beyond identical sequence contexts, we created a purely sequence based DNA methylation predictor which can be applied to all the CpGs within PMDs. To this end, we generated a position specific linear dinucleotide model (see Materials and Methods) and trained it on 100K (out of 6.1M) single CpG methylation data points. The accuracy of the model on an independent set of 100K data points was 31%, corresponding to a correlation of r = 0.56 between the predicted and the actual methylation data (Figure 4a). While this is less striking than the correlation of r = 0.8 between IMR90 and FF, it still provides additional evidence that DNA sequence most likely provides the information required to set the methylation status of a given CpG. This signal is PMD-dependent, as performing the same analysis in H1 which contains virtually no PMDs results only in a correlation of r = 0.20. The model furthermore allows us to infer the relative importance of the various sequence features (Figure 4b). The most informative feature is CpG content +−20 bp around the central CpG. This means that the methylation status of a CpG depends on the presence of other CpGs in its environment. The two CpGs immediately surrounding the central CpG have an exceptionally high impact on methylation. Outside of the +−20 bp band the impact of CpGs decreases substantially. The level of methylation generally rises with increasing numbers of CpGs. Interestingly this is the opposite of what occurs in CpG islands where high CpG density coincides with strongly reduced methylation levels [1]. Furthermore, various dinucleotides immediately flanking the CpG show positive as well as negative contributions. While TT and AA negatively influence the methylation levels on both sides, TG, CT, CA and AG have opposite contributions on either side of the CpG. Finally, various dinucleotides show a more subtle but clearly periodic signal of 10 bp reminiscent of the DNA helix turn. This again suggests that the nucleosome might play a role in determining methylation levels in PMDs. From this analysis it is however unclear to what extent methylation levels can be explained by positioned nucleosomes.

To investigate whether similar sequence preferences exist in other PMD-containing tissues, we trained our dinucleotide model on HMEC and colorectal cancer cells (Figure 4c). This revealed consistent sequence features across all PMD-containing methylomes. Additionally, applying the model to the CpGs outside of PMDs showed that these sequence features are PMD-specific. As a control, we trained the model on H1 using the IMR90 PMD annotation (as the tissue itself does not contain PMDs). This analysis did not reveal any clear sequence preferences (Figure 4c). In accordance with these findings, the respective models can predict methylation levels within PMDs with good accuracy, but fail to do so outside of PMDs and in methylomes without PMDs (Figure 4d). We conclude that the high variability of methylation levels in PMDs are, to a significant extent, determined by specific DNA sequence features that are conserved across diverse cell types.

### Validation of the dinucleotide features

Allele-specific analysis provides a powerful tool to validate our inferred dinucleotide features. The dinucleotide model suggested that the change of a particular dinucleotide in the vicinity of a CpG can influence its methylation level. Heterozygous SNPs close to a homozygous CpG should therefore result in differences in methylation between the CpGs of the two alleles. Bisulfite reads which overlap both the SNP and the CpG in the vicinity can be uniquely assigned to one allele. This does not only allow us to calculate methylation levels for the CpG on both alleles separately, but also to compare the difference in methylation to the nucleotide variation at the heterozygous SNP (see Material and Methods). Starting from a total set of 647K heterozygous SNPs in IMR90, we found 56K CpGs in PMDs which were present on both alleles, had exactly one single SNP within a window of +−40 bp (see Materials and Methods) and showed a coverage of at least 10 bisulfite reads for both alleles. From this dataset we inferred the contribution of each dinucleotide, at each distance, to the measured methylation difference between the two alleles (see Materials and Methods). Figure 4e displays the results in the same fashion as the previous non-allele specific analysis (Figure 4b). Side by side comparison shows that the sequence features obtained from the two different analytical approaches are highly similar, in particular the central role of the CpG density and the various dinucleotides immediately flanking the central CpG. The periodic 10 bp signal is not as clear as before but this is likely due to the limited number of data points available for allele-specific analysis.

### Nucleosome positioning explains only a limited amount of variation in methylation in PMDs

**Figure 5:**
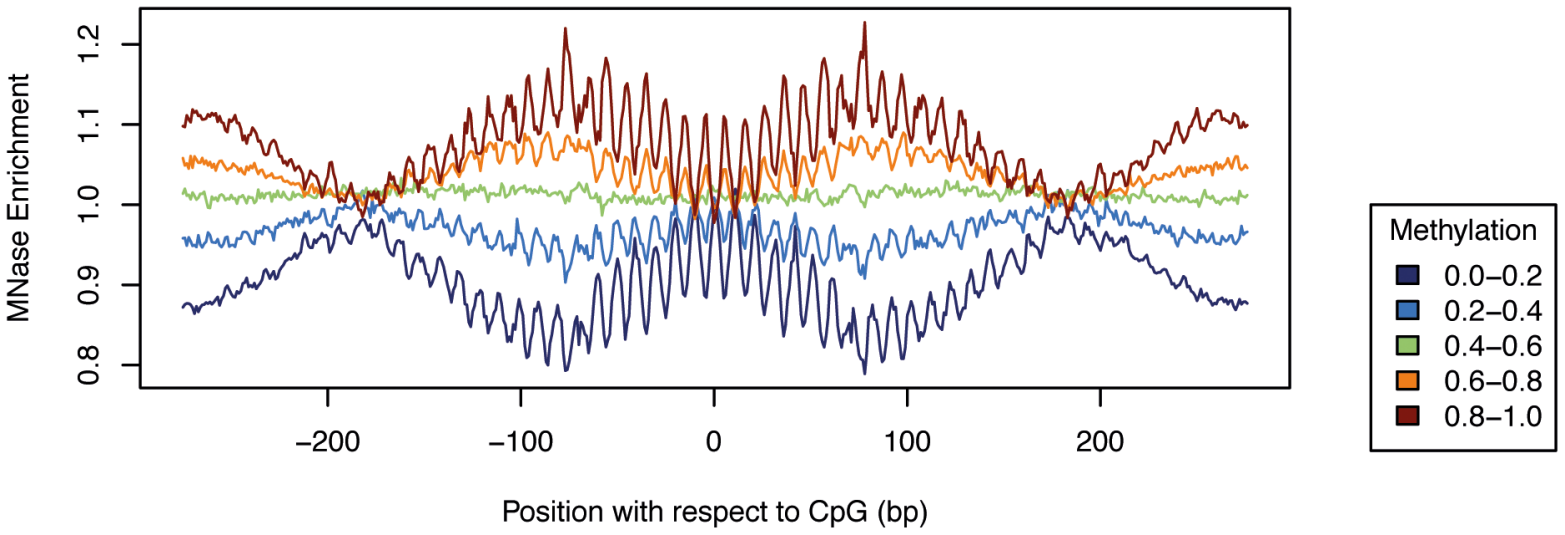
Nucleosome positioning explains only limited variation in methylation in PMDs. Composite MNase profiles in IMR90 for CpGs within PMDs stratified by methylation level.

In order to relate methylation levels to positioned nucleosomes, we created a high coverage nucleosome map in IMR90 by Micrococcal nuclease (MNase) treatment and sequencing of mononucleosomal DNA. We obtained a total of 517 million uniquely mapping reads, which is comparable to the amount generated in previous high coverage nucleosome studies [20]. To determine if nucleosome positioning shows methylation dependent patterns in PMDs, we stratified the CpGs according to their methylation levels into 5 equally spaced bins and created composite MNase profiles for each individual bin (Figure 5). The profiles show that methylated CpGs within PMDs overlap less frequently with positioned nucleosomes (180 bp periodicity) and tend to lie on a single side of the double helix (10 bp periodicity). Others have previously analyzed the relationship of methylation and nucleosome positioning outside of PMDs and have found either decreased [22] or increased methylation at positioned nucleosomes [19]. Although our data gives support to the former finding, Figure 5 also shows that the MNase enrichments (compared to the average over all CpGs) are very small (1.2 fold). This suggests that nucleosome positioning may have only little predictive power. To test this we performed a linear regression using positional MNase read counts (centered around the CpGs) as predictors and methylation levels as the response variable. This resulted in a correlation of only r = 0.15, substantially less than the performance of the nucleotide model (r = 0.56). The correlation of 0.15 is an upper bound to the potential explanatory power of the MNase signal and might even be substantially lower due to possible confounding factors such as the cutting preferences of the MNase enzyme or sequencing biases [23]. We thus conclude that nucleosome positioning plays only a minor role in determining the exact methylation levels in PMDs. Sequence features, in particular the local CpG density provides substantially more information about the methylation status.

## Discussion

PMDs have been characterized as long domains (mean = 153 kb) with decreased average methylation [5]. In this study, we showed that in IMR90, this overall decrease is accompanied by a strong increase in the variability along the genome, with methylation levels ranging from 0% to 100% in a roughly uniform fashion. These seemingly disordered methylation patterns however are conserved across cell types at the single CpG level suggesting a mechanism that provides high local specificity. Using comparative sequence analysis as well as a positional dinucleotide model, we showed that outside of CpG islands and DHS specific DNA sequence features contribute to the methylation levels observed in PMDs. These include CpG density as the main driving feature followed by various dinucleotides immediately flanking the CpG, as well as a minor contribution from sequence preferences reminiscent of the sequence preference of positioned nucleosomes. CpG density, in particular, showed the strongest contribution in a +−20 bp band, with an exceptionally high impact of the two CpGs immediately surrounding the central CpG. This may suggest cooperative behavior on two distinct levels. In the case of two adjacent CpGs, the DNA methyltransferase (DNMT) depositing the methylation mark on the first CpG might directly methylate the neighboring CpG whereas in the case of a wider distance, cooperativity might be caused by a DNMT sliding along the DNA, or the recruitment of an a additional DNMT depositing the second mark. Importantly, CpG density is positively correlated with methylation levels and thus promotes methylation. An early indication of this finding, without a link to PMDs, has been given by [24] and is, interestingly, the opposite to what has been shown for CpG islands [25]. We additionally investigated the role of nucleosome positioning by creating a high coverage map in IMR90. While we provide evidence for a slight decrease in methylation at positioned nucleosomes, we concluded that nucleosome positioning plays only a minor role in determining the exact methylation levels in PMDs. Sequence features, in particular the local CpG density provided substantially more information about the methylation status.

In embryonic stem cells, the methylation machinery maintains a fully methylated state in most of the genome. In fibroblasts however, large domains covering 35% of the genome show reduced levels of methylation (PMDs). This suggests that the DNMTs are impaired in their capacity of maintaining the fully methylated state at those domains. Interestingly this loss in methylation, on the level of single CpGs, reveals a methylation pattern that is not apparent in all cell types. We speculate that this could be caused by a reduced access of the methylation machinery in PMDs. As this would lead to a reduced effective concentration, there might be a critical point where the concentration of the DNA methylation machinery becomes rate limiting for the process of methylation. In this case, the intrinsic sequence preferences might surface whereas in a normal configuration, these patterns would be overwritten and converted into a fully methylated state. Our dinucleotide model provides indirect evidence for this hypothesis, as it shows that the methylation status of a CpG in PMDs depends on the presence of other CpGs in the vicinity. This cooperative aspect of the methylation machinery would result in increased methylation levels in genomic regions with higher CpG density. A recent experimental study in a mouse lung-cancer model [26] suggests that the de novo methyltransferase Dnmt3a may be an important player in this process as it is required to maintain the fully-methylated state in active regions. It could thus be hypothesized that it is the reduced access of Dnmt3a to PMDs which leads to the reduction of methylation.

The relationship between methylation and DNA sequence features has been extensively investigated in the past. These studies were either performed on limited sets of CpGs for which methylation measurements were available [27], [28] or with a particular focus on CpG islands. The latter methods were initially based on sequence alone [25], [29] and were later augmented with epigenetic data [30], [31],[32]. The analysis presented here departs from these earlier studies by focusing on the large majority of CpGs that lie outside of CpG islands and by explaining the previously unknown and unexplained extensive variability in methylation levels within PMDs. The relationship between methylation levels outside of CpG islands and specific sequence features irrespective of PMDs has been investigated in a recent study about allele-specific methylation in mouse [33]. The authors identified sequence motifs enriched in the immediate vicinity (+−2 bp) of CpGs methylated in an allele-specific fashion. Although the reported sequence features are comparable to our findings, they differ in the extent of the sequence environment (+−2 bp compared to +−20 bp) and thus differently assess the contribution of the CpG dinucleotide in the wider sequence context. It is however intriguing that when projecting the inferred motifs to various human methylomes, they see highest agreement in the tissues containing PMDs. This supports our finding that sequence features are much more predictive of methylation levels inside versus outside of PMDs.

Due to the ever-decreasing costs in sequencing, researchers can now generate large numbers of single-base methylomes in a variety of conditions. PMDs occur in only a subset of conditions but if present can cover up to 40% of the genome. This will inevitably lead to a large number of differentially methylated CpGs. Here we provide a sequence-based explanation for the methylation differences in PMDs and suggest that these should be treated separately from the changes outside of PMDs. This study adds to the growing body of literature that aims at disentangling the wealth of information contained in base-pair resolution methylation maps and should thus be of great importance for the interpretation of the large number of methylomes that will be generated in the nearby future.

## Materials and Methods

### Data processing for the various methylomes

Unless stated differently, all methylomes in this study have been processed as follows: Methylaton levels from both strands of a given CpG were combined. CpGs in CpG islands (www.genome.ucsc.edu) were removed after extending the CpG islands by 100 bp. CpGs overlapping DHS were removed as well for the cell types H1 and IMR90 for which DHS datasets were available. Only autosomal CpGs with a minimum coverage of 10 were considered. All CpGs overlapping a SNP from dbSNP (09.11.10) were removed to ensure that the methylation levels are not cofounded by polymorphisms. We used the software MethylSeekR [18] to detect PMDs in the various methylomes and created a high confidence set by considering only the PMDs with a length of least 200 kb. CpGs outside PMDs were defined as the CpGs, which did not overlap any PMD. For Figure 1, PMD coordinates from the original publication [5] were used to avoid circular inference of the uniform distribution of methylation levels in PMDs. MethylSeekR makes explicit use of the distribution of methylation levels while in the original publication PMDs were only defined by a reduction in mean methylation. Classification of the CpGs into repeat classes was done based on UCSC (genome.uscsc.edu) repeat annotation using the repClass column in the rmsk table. All analyses throughout the manuscript were performed in R (www.r-project.org) using core packages from bioconductor [34].

### Positional dinucleotide model

To predict methylation levels from DNA sequence, we created a linear regression using dinucleotide features as predictors and the measured methylation level as the response. For each CpG in consideration, the sequence environment (+−78 bp) was extracted and split into non-overlapping blocks of dinucleotides. Each dinucleotide was interpreted as a categorical variable with 16 states (15 dummy variables in the regression). Thus in total the regression contained 78*15 = 1170 variables. For visualization as a heatmap, the left out dinucleotide was introduced back (as a zero) into the list of coefficients. These were then normalized to mean = 0. Training and testing was performed on separate sets of 100K data points selected randomly.

### Positional dinucleotide model to predict allelic differences in methylation

To create an allele specific methylation map for the IMR90 cell lines, we downloaded the mapped reads, a list of SNPs in IMR90 (see datasets) and realigned the reads using the bioconductor package QuasR (www.bioconductor.org/packages/2.12/bioc/html/QuasR.html). From the list of SNPs, two separate genomes were produced (Reference and Alternative). After C to T conversion of the reads and the genomes, alignments were performed using bowtie [35]. Only unique mappers with at most three mismatches were considered. The two separate alignments were combined into one, by flagging reads mapping equally well to both genomes as “Unknown” and reads mapping better to one genome than to the other as “Reference” or “Alternative”. Using this flag, methylation levels for CpGs were calculated separately for the Reference and the Alternative allele. Only CpGs covered by at least 10 bisulfite reads in both alleles were considered for further analysis. From a total set of 647K heterozygous SNPs in IMR90, we found 56K CpGs in PMDs which were present on both alleles and had exactly one single SNP within a window of +−40 bp. From this dataset, we inferred the contribution of each dinucleotide to the difference in the methylation between the alternative allele and the reference allele (dm = m_A-m_R). Since each CpG contains only one SNP in its environment, inference can be performed for each position independently. However since a change in sequence involves two dinucleotides (one on each allele), the values for delta methylation dm need to be interpreted as the change in methylation caused by converting a particular dinucleotide to another one. This relationship is described by the equation dm = s_d(A)–s_d(R), where s_d(A) represents the contribution of the dinucleotide present in the alternative genome and s_d(R) represents the contribution for the dinucleotide in the reference genome. Since there are multiple data points, this leads to a linear regression (no constant term) with 16 variables and as many equations as there are SNPs at a particular position (between 538 and 1942 in this dataset). This regression however is singular as an arbitrary constant can be added to the coefficients without having any effect on the quality of the fit. We thus set the dinucleotide TT arbitrarily to zero and used it as a reference. Thus the final regression consisted of 15 variables and was performed independently for each position. For visualization as a heatmap, the coefficients (including a value of zero for TT) were mean normalized.

### Mononucleosome-BisSeq (MN-BisSeq) library preparation

Chromatin isolation from IMR90 was performed under native conditions as described [36]. MNase treatment was performed with 5 U (Roche Nuclease S7, catalog number 10107921001) at 37°C for 30 min per 1 million cells. Digestion was tested on 2% agarose gel resulting in the highest proportion of mononucleosomal fraction. Mononucleosomal fraction was then extracted from the agarose gel using QIAquick Gel Extraction Kit (Qiagen). Library preparation was performed using Illumina Genomic DNA Sample Preparation Guide starting with 2 µg of mononucleosomal fraction DNA. Single End Genomic Adapter Oligo Mix was used and the library was amplified for 10 cycles using Illumina PCR primers 1.1 and 2.1. Final PCR product was purified using Agencourt AMPure XP beads (Beckman Coulter). Quality of the libraries and template size distribution were assessed by running an aliquot of the library on an Agilent 2100 Bioanalyzer (Agilent Technologies).

### Analysis of the MNase data

A total of 697M single-end reads were mapped to hg18 using bowtie [35]. 517M uniquely mapping reads were considered for further analysis. Given the footprint of 147 bp of the nucleosome, the reads were shifted by +/−74 bp dependent on the strand. Reads starting exactly within the interrogated CpGs (before shifting) were not considered as we observed strong discontinuities of the MNase signal when creating average profiles with respect to a central CpG. We assumed that this is caused by sequence specific biases of the MNase treatment and thus decided to remove the critical alignments. The raw data as well as processed files were submitted to GEO (www.ncbi.nlm.gov/geo) and can be retrieved using the accession GSE44985.

### Publicly available datasets used in this study

Methylomes for H1, IMR90, IMR90-iPSC and FF as well as the mapped reads from IMR90 (used for the allele specific analysis) were downloaded from http://neomorph.salk.edu/ips_methylomes/data.html. The Colorectal cancer methylome was downloaded from http://epigenome.usc.edu/publicationdata/berman20101101/. IMR90 SNPs were downloaded from http://www.genboree.org/EdaccData/SBS-SNPs/IMR90.Methyl-C.Hg18.SNPs/. The HMEC methylome was downloaded from GEO (accession number GSM721195). The DNase I hypersensitive sites for IMR90 and H1 were downloaded from the ENCODE Consortium data repository at genome.ucsc.edu/ENCODE (narrowPeak lists). All datasets were provided in coordinates for the hg18 human genome assembly.
